# Innovative Construction Materials for Sustainable and Greener Applications

**DOI:** 10.3390/ma19101915

**Published:** 2026-05-07

**Authors:** Yongfan Gong, Chenhui Zhu

**Affiliations:** 1 College of Civil and Transportation Engineering, Yangzhou University, Yangzhou 225127, China; 2 School of Transportation and Civil Engineering, Nantong University, Nantong 226200, China

As the volume of industrial solid waste and construction and demolition (C&D) waste continues to rise, along with a growing societal focus on efficient resource utilization and environmental protection, research on the application of solid waste in building materials has become increasingly vital [1]. It is imperative to thoroughly explore the physicochemical properties, interfacial behavior, and long-term durability of diverse types of solid waste, including metallurgical slag [2], fly ash [3], and recycled concrete aggregates [4], during the preparation of building materials. This exploration is of great significance in achieving high-value utilization of solid wastes in sustainable construction materials, optimizing mix proportions, and ensuring the structural safety and longevity of engineering structures throughout their service life. This Special Issue (SI), “Innovative Construction Materials for Sustainable and Greener Applications”, presents recent theoretical, experimental, and applied research findings regarding the resource utilization of solid waste and the application of low-carbon concrete. This Editorial summarizes the nine publications (eight research articles and one review article) included in this SI.

In contrast to the conventional production of building materials, which primarily hinges on natural sand, gravel, and cement, the incorporation of solid waste into building materials exhibits prominent features of resource recycling and environmental synergy. This endows it with distinctive advantages in curbing carbon emissions, conserving natural resources, and alleviating the strain on solid-waste landfill sites [5]. Consequently, high-value applications of solid waste in innovative construction materials for sustainable development have attracted extensive research attention in recent years [6]. Zong et al. [7] reviewed the applications and mechanisms of admixtures and industrial by-products in cement-based self-leveling mortars, noting that China’s standards are more detailed. They also found that higher-molecular-weight admixtures performed better and that industrial by-products reduced emissions and resource consumption, with by-product gypsum showing the greatest potential. Yan et al. [8] designed a novel self-compacting recycled pervious concrete that resolved the challenge of balancing mechanical strength and permeability in traditional pervious concrete. The material demonstrated superior sulfate resistance and permeability, making it more suitable for environments containing Na_2_SO_4_.

Multi-source solid-waste cementitious materials exhibit the potential to fully replace cement through synergistic complementation and complex salt effects. These materials are particularly applicable to scenarios such as road base construction, mine backfilling, block brick production, soil stabilization, and non-structural precast components. Multi-source solid-waste cementitious materials integrate the advantages of waste utilization with cost-effectiveness, delivering remarkable benefits and representing a promising direction for the development of sustainable building materials. Zhang et al. [9] successfully developed a full solid-waste-based cement utilizing coal gangue, carbide slag, and desulfurized gypsum as raw materials. The compressive strength of the concrete reached 35.01 MPa after 6 h, which was 48.6% higher than that of sulfur-aluminate cement concrete. Doo-Yeol Yoo et al. [10] developed a completely cement-free ultra-high-ductility strain-hardening slag composite, with the optimal formulation achieving a tensile strength of 10.05 MPa and an ultra-high tensile strain of 9.19%. Moreover, carbon emissions were reduced by 70.2% compared to traditional cement-based strain-hardening composite materials. Xian et al. [11] successfully developed a novel fully solid-waste cementitious material through accelerated carbonation curing technology, utilizing steel slag and water-quenched slag as raw materials. This material not only attained a maximum CO_2_ capture rate of 13.2% and achieved a 28-day compressive strength of up to 57.2 MPa but also exhibited excellent photocatalytic degradation performance. By employing circulating fluidized bed fly ash, granulated blast furnace slag, steel slag, and flue-gas desulfurization gypsum, Liu et al. [12] prepared a multi-source solid waste cementitious material that exhibited a significantly increased 28-day compressive strength of 56.2 MPa and a carbon footprint merely one-tenth that of cement, thereby offering technical support for the large-scale resource utilization of industrial solid waste. Li et al. [13] proposed a low-alkali, fully solid-waste cementitious material, composed mainly of ammonia-soda residue, steel slag, and granulated blast furnace slag, for preparing cemented tailing backfill (CTB). CTB meets the workability and strength requirements when the tailings-to-sand ratio is more than 0.5 and the water-to-binder ratio ranges from 0.9 to 1.1. Compared with cement-based CTB, this material reduces energy consumption by 62.73%, CO_2_ emissions by 89.3%, and cost by 52.4%, demonstrating significant environmental and economic advantages.

Nowadays, with the swift advancement of green building material technology, a growing array of innovative building materials tailored to sustainable and eco-friendly applications has been developed, encompassing solid-waste-based materials [14], recycled concrete systems [15], and low-carbon cementitious materials [16]. Future research endeavors should place greater emphasis on assessing the long-term durability and environmental adaptability of these novel materials within their service environments. Concurrently, given that the environmental synergistic behaviors of materials are intricately linked to their microstructural evolution, a broader application of multiscale analysis methods is imperative for in-depth investigation. Furthermore, the integration of artificial intelligence (AI) technology into green material design, performance forecasting, and mix proportion optimization [17,18] also warrants thorough exploration.
